# The impact of low-dose gamma radiation on immune modulation in a mouse model of spontaneous mammary gland tumorigenesis

**DOI:** 10.3389/fimmu.2025.1635779

**Published:** 2025-11-03

**Authors:** Abrar Ul Haq Khan, Melinda Blimkie, Bryan Marr, Jin Wu, Tyler Pack, Shelby Kaczmarek, Dong-Hyeon Jo, Holly Laakso, Seung-Hwan Lee

**Affiliations:** ^1^ Department of Biochemistry, Microbiology, and Immunology, Faculty of Medicine, University of Ottawa, Ottawa, ON, Canada; ^2^ The University of Ottawa Centre for Infection, Immunity, and Inflammation, Ottawa, ON, Canada; ^3^ Ottawa Institute of Systems Biology, University of Ottawa, Ottawa, ON, Canada; ^4^ Radiobiology and Health Branch, Canadian Nuclear Laboratories Ltd., Chalk River, ON, Canada

**Keywords:** LDR, NK cells, NKG2D, inflammation, tumor, LNT

## Abstract

Understanding the impacts of low-dose ionizing radiation exposure has significant public health implications. However, the effects of low-dose ionizing radiation on immune modulation and cancer progression remain contentious. This study aimed to investigate the impact of chronic low-dose gamma radiation on mammary tumorigenesis and immune homeostasis using a transgenic mouse model. Female MMTV-neu transgenic mice were exposed to continuous whole-body ^60^Co gamma radiation over a period of 56 days, thereby receiving cumulative absorbed doses of 10, 100 and 2,000 mGy. Mice were analyzed at 3.5, 6 and 8 months of age for changes in immune cell composition and function, as well as tumor development. We found that mice exposed to LDR exhibited transient increases in NK cell frequency, along with improved IFN-γ production following *ex vivo* stimulation. Notably, the expression of NKG2D on NK cells was upregulated following LDR exposure. Low-dose radiation also modulated inflammatory cytokine profiles and immune cell populations, such as macrophages and myeloid-derived suppressor cells. Despite these immune changes, the overall impact on tumorigenesis was minimal. Although our data indicated that the LDR treatment did not impact survival and cancer progression, the observed results of NK cell proportion, activation and function provide evidence of the stimulatory effects of LDR on NK cells. These findings aim to contribute to health risk assessments and advise radiation protection regulations.

## Introduction

The effects of low-dose radiation (LDR) on biological systems remain a subject of intense scientific debate and investigation. Low-dose ionizing radiation is commonly encountered via medical diagnostics, occupational exposures, and environmental exposures, thereby posing potential public health risks that are not fully understood. A comprehensive understanding of LDR-induced effects is essential for establishing effective safety standards and optimizing its potential therapeutic benefits while minimizing risks.

Substantial evidence links high-dose radiation (HDR) exposure with detrimental biological effects, including increased cancer risk and immunosuppression (1–3). On the other hand, the effects of LDR exposure remain an active area of research. LDR refers to absorbed doses under 100 mGy and dose rates under 5 mGy/hour (4, 5). According to the linear-non-threshold (LNT) model, the health risks of radiation are linearly related to dose, with no threshold beneath which risk is eliminated (6). The accuracy of this model is contested, as ongoing research seeks to better characterize the health risks from LDR exposure and determine whether risk thresholds or hypersensitivities exist. Others have proposed a hormesis model in which LDR provokes beneficial effects such as activation of anti-tumor immunity, cellular antioxidant responses, reduced cancer risk, increased lifespan and protection from subsequent radiation exposures (7–13).

The major risk of radiation exposure is cancer (14, 15). Numerous reviews and meta-analyses of the low-dose radiation epidemiological literature have found evidence for increased cancer risk in the low-dose range. There is also experimental evidence to support radiation carcinogenesis in the low-dose range (16–18). The National Council on Radiation Protection 2017 report found that epidemiological evidence supports the continued use of the LNT model for assessing risk and protection measures (19). However, others have challenged these perspectives, citing evidence where cancer risk was absent or non-linear and opposing the interpretations of the epidemiological literature (8, 10, 20–26). Furthermore, the hormesis model has been used to describe studies in which LDR was associated with reduced cancer risk or therapeutic effects, suggesting a more complex interaction between low-dose radiation and biological systems than the LNT model can account for (9, 13, 27, 28).

The immune system’s response to low-dose radiation is complex and poorly understood. An excellent review of this topic was completed by Luminesky et al. (12). Epidemiological, clinical, and experimental studies suggest that low-dose radiation exposure can fundamentally and durably reshape the immune system. This includes transient and persistent changes in the proportion and phenotype of immune cell populations as well as systemic alterations in cytokines and immunoglobulins. However, the results are often complicated because LDR can influence immune parameters in diverse and sometimes opposing ways depending on factors such as total dose, dose rate, cell type, genetic background, and overall health status. More work in this field is vital to characterize the consequential and therapeutic implications of LDR-induced immunomodulation.

One critical area of study regards understanding how LDR impacts immunity in the context of cancer. It is important to question whether the increased risk of cancer found in certain epidemiological studies is driven at least in part by immunomodulation. Furthermore, there is a lack of experimental research describing immune parameters in models of LDR-induced carcinogenesis. On the other hand, certain studies have implicated LDR in enhanced immune surveillance and anti-tumor responses (29–34). *In vitro* stimulation of immune cells has also been explored to improve anti-tumor immunity with immunotherapeutic potential (35–39).

There remains little understanding as to whether the impact of LDR on the immune system affects tumorigenesis. Most of the available data is acquired with inadequate methodological approaches while displaying insufficient statistical significance (40–43), raising substantial queries. Furthermore, research into gamma radiation-induced changes in the cellular immune response, with respect to mammary tumor development, has been limited. To address this gap, we utilized an *in vivo* mouse model to investigate the effects of chronic low-dose gamma radiation on the development and progression of mammary cancer by using a transgenic mouse model (FVB/N-Tg(MMTVneu)202Mul/J). These mice are characterized by the over-expression of the *neu* gene, a rat homolog to the human *HER2* gene, leading to the spontaneous development of mammary adenocarcinomas with a mean tumor latency of 7.5 months in these mice. The first tumor appears as early as 4 months of age, with 75-80% of mice developing lung metastases by the age of 7–8 months (44–46). During this study, ^60^Co was used as the gamma-rays source in the radiation facility at Canadian Nuclear Laboratories (CNL), which is uniquely designed to accommodate the exposure of experimental animals. Moreover, this state-of-the-art facility enables precise irradiation of animals from low to high doses over an extended period of time. To the best of our knowledge, this is the first study that investigates the effects of gamma radiation on the immune system in an *in vivo* model of spontaneous tumorigenesis.

## Methodology

### Mice

A total of 455 female FVB/N-Tg(MMTVneu)202Mul/J mice (hereon denoted as MMTV-Neu; Stock # 002376, Jackson Laboratories) were bred from the frozen embryo, then purchased at 4 weeks of age and entered the study at 6 weeks of age. Mice were housed in the Specific Pathogen-Free Biological Research Facility (BRF) at CNL. Six mice were housed per cage in individually ventilated Thoren cages with ad-libitum access to food (Charles River Rodent Chow 5075) and reverse osmosis (RO) water. Mice were externally exposed to chronic gamma rays (^60^Co) in the BRF Gamma Beam Hall for total absorbed doses of 0 (UT), 10, 100, or 2,000 mGy over 56 days. The 2,000 mGy was used as a control high dose. All animal husbandry and experimental procedures were approved by the Canadian Nuclear Laboratories Animal Care Committee in accordance with the standards of the Canadian Council on Animal Care (CCAC).

**Figure 1:**
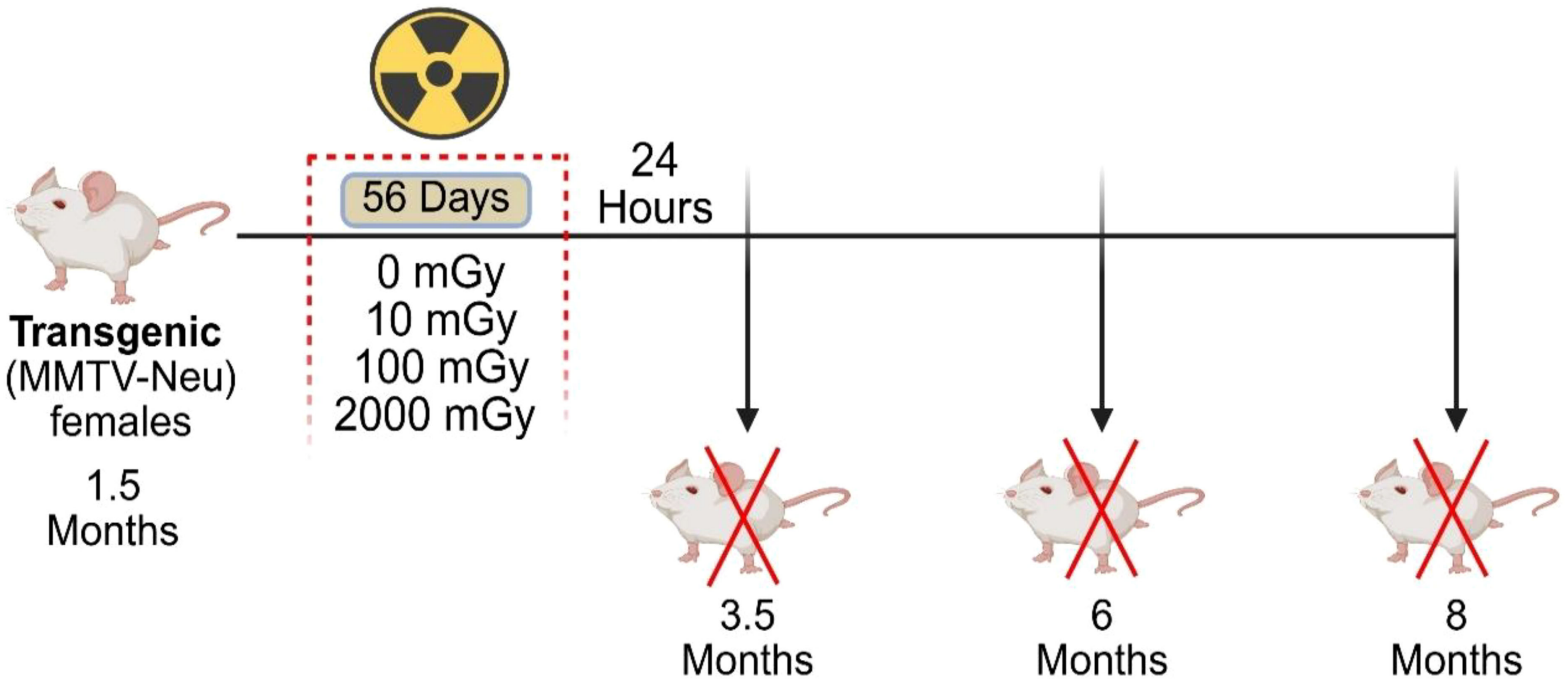
Schematic representation of the study. Transgenic “MMTV-Neu” female mice underwent chronic gamma radiation exposure, starting at 1.5 months of age, to total doses of 0, 10, 100, and 2000 mGy over 56 days. Mice were sacrificed at three time-points: 3.5, 6, and 8 months of age. Blood, spleen, lungs and mammary glands were collected for further processing and analysis. Tumor number and volume were assessed in tumor-bearing mice at the time of sacrifice.

The experimental endpoints were 3.5, 6, and 8 months of age. These endpoints were chosen to examine tissues at multiple stages of mammary tumor development and post-exposure response; before tumor development (3.5 months; immediately following irradiation); during tumor development (6 months; 2.5 months post-exposure); and high tumor burden/metastasis development (8 months; 4.5 months post-exposure), as shown in **Figure 1**. Mice were euthanized via exsanguination under Isoflurane gas anesthesia, followed by cervical dislocation. Blood, mammary glands, lung, spleen, and tumor samples were collected from 455 mice for further processing and analysis.

### Gamma hall dosimetry and exposure

At 1.5 months of age, MMTV-Neu mice were exposed to an open beam ^60^Co source (Gamma Beam 150C; Nordion) in the 30m-long Gamma Beam Irradiation Facility. The ^60^Co source had a total activity of 2.97 Ci (as of May 2022). Due to the disparity in dose rates between the low dose (10 mGy and 100 mGy) and the high dose (2,000 mGy) groups, the mice were irradiated in separate batches. The low-dose cohorts were irradiated with 1.75’ of lead shielding. Both physical shielding and the distance of the animal cages from the primary source allowed the required dose rates of 7.7 µGy/h (for the total absorbed dose of 10 mGy) and 77 µGy/h (100 mGy). The high-dose cohort did not require shielding during irradiations; mice received an unshielded dose of 1.49 mGy/h (absorbed dose of 2,000 mGy). Dosimetry was completed using Exradin^®^ A8 ion chamber and SuperMax™ electrometer readings to determine average gamma radiation dose rates in the hall. Theoretical Monte Carlo N-Particle Transport Code (MCNP) modelling of the shielded source estimated that 61-64% of the total dose originated from pure ^60^Co where the remaining contributions were from wall scatter (unpublished data; CNL internal technical memorandum). During dosimetry measurements and during the 56 days of exposure, passive dosimeters containing Harshaw thermoluminescent dosimeter TLD-100e LiF: Mg, Ti chips (Harshaw Chemical Co., Solon, OH) were placed in empty mouse cages distributed at the corners and centers of each animal rack to record total absorbed gamma dose. Average dose rates for gamma radiation during the animal exposure period were derived by dividing the total gamma dose, determined by the mean TLD measurement per animal rack, by the total exposure period (adjusted for beam downtime necessary to accommodate animal husbandry activities). While in the gamma beam hall, mice were housed in wooden racks in Thorene cages, at 23-24 °C, 37-40% humidity, and an atmospheric pressure of 754–759 mmHg.

### Blood analysis

Mice were anesthetized using Isoflurane gas and then peripheral blood was taken via cardiac puncture at the time of euthanasia and kept refrigerated under continuous rocking/shaking for no longer than 1-hour post-collection. Blood samples were analyzed on Zoetis Vetscan MS5 hematology analyzer. To collect plasma, whole blood was centrifuged at 12,000 × g for 10 minutes at 4 °C. Plasma was carefully transferred to 1.5 mL Eppendorf tubes, snap-frozen in liquid nitrogen and subsequently stored at -80 °C.

### Cytokine analysis

Blood plasma samples stored at -80 °C were thawed on ice, and aliquots of 50 µL were collected for analyses. The concentration of select cytokines, chemokines, and growth factors in the plasma was analyzed using BioPlex 200 system and Bio-Plex Pro™ Mouse Cytokine 23 plex panel, Group I (Bio-Rad, Hercules, California, USA). Each plate was designed and assembled according to the manufacturer’s instructions. Briefly, antibody coupled magnetic beads were incubated with standards and plasma samples. This was followed by incubation with biotinylated conjugate antibodies, and lastly incubation with streptavidin phycoerythrin (SA-PE) conjugate antibodies. Beads were washed using the Integra Viaflo Assist automated multi-channel pipette (Mandel Scientific, Canada) after each incubation to remove any unbound components. Two different dilutions of standards were run at the customary dilution factor of 4 and at a dilution factor of 40. Each plate was run twice on the BioPlex 200 array reader, on both the low photomultiplier (PMT) and high PMT settings with the appropriate standard selected. The cytokines were detected with a dual laser system: The green “reporter” laser (532 nm) and the red “classify” laser (635 nm) excite the PE dye and dyes inside the magnetic beads, respectively, to allow high throughput detection of all 23 cytokines at once. Data was analyzed using the BioPlex Manager™ 6.1 software. The standard curves were modelled using a 5-parameter logistic (5PL) regression, and cytokine concentration results were presented in pg/mL. Each sample was run in duplicate on different plates and two samples were consistently run on every plate to function as inter-plate controls; these controls allowed for normalization between plates and runs.

### Single cell isolation

Spleens were harvested promptly following euthanasia, and a single-cell suspension was generated using the following process: dissociating tissues using a 3 ml syringe plunger and passing through a 70-μm filter (Bio Basic Canada Inc.), centrifugation at 1200 rpm for 10 min at 4 °C; and finally, washing with RPMI containing 2% fetal bovine serum (FBS) (Gibco™, Canada). The cell pellet was reconstituted in 1 mL of red blood cell lysis buffer (Roche) immediately followed by vortexing. Cells were washed with RPMI containing 2% FBS and filtered using a nylon mesh before counting. To obtain leukocytes from lungs, tumors, and mammary glands, tissues were dissociated into small pieces using dissection scissors and then mixed with extraction buffer (RPMI-10% FBS) containing 25U/mL collagenase VIII (Sigma). Sample homogenization was done at 37 °C using gentleMACS™ Dissociator (Milteney biotech, USA) followed by straining with a 70-μm filter. Lymphocyte isolation was performed using Percoll gradient centrifugation (Percoll^®^, Millipore Sigma) according to the manufacturer’s instructions. Cells were filtered through a nylon mesh before counting. All the assays were performed on splenic lymphocytes; other tissues were used only for immune profiling.

### *Ex vivo* functional assay

For *ex vivo* NK cell intracellular IFNγ measurements, freshly derived spleen leukocytes were stimulated with either a combination of IL-2 (100U/ml, obtained from NCI Preclinical Repository, USA) and IL-12 (10 ng/ml) (eBioscience™), or with plate coated anti-NKp46 (BioLegend™) for 1 hour and then incubated in RP-10 media containing 5 μg/ml brefeldin A (Invitrogen™) for 4 hours, followed by intracellular staining. For *ex vivo* T cell intracellular IFNγ measurements, freshly derived spleen leukocytes were stimulated with anti-CD3/28 for 16 hours and then incubated in RP-10 media containing 5 μg/ml brefeldin A (Invitrogen™) for a further 4 hours, followed by intracellular staining.

### Antibodies and flow cytometry

Single-cell suspensions (1×10^6^ cells) were incubated at 4 °C for 10 min with α-CD16/32 (clone 2.4G2, from Bioexpress (USA)) to reduce non-specific binding. Cells were labelled with various combinations of fluorochrome-conjugated monoclonal antibodies (mAbs) and incubated at 4 °C for 25 min. The following mAbs were used: anti-TCRβ (H57-597), anti-CD8 (53-6.7), anti-CD49b (DX5), anti-IFNγ (XMG1.2), anti-CD11b (M1/70), anti-NKG2D (CX5) from eBioscience™; anti-CD19 (1D3), anti-CD4 (RM4-5), anti-F4/80 (T45-2342), anti-CD69 (H1-2F3), anti-Ly6C (AL-21), anti-Gr1 from BD Biosciences™, anti-CD43 (1B11) activation- Glycoform from BioLegend™ and Live/Dead Fixable Yellow Dead Cell Stain from Invitrogen™. The intracellular staining of IFNγ was performed using Cytofix/Cytoperm protocols (BD Biosciences™). Cells were acquired using Thermofisher Attune NxT flow cytometers, and data was analyzed using Kaluza 1.3 Analysis software (Beckman Coulter) or FlowJo (V10).

### Tumor measurement

Tumors were measured weekly using digital calipers, both noninvasively over the skin of live mice during the study and at the time of post-mortem dissection. Mice with cumulative tumor volumes greater than 2.9 cm^3^ were euthanized at any point in the study, as per the clinical endpoints determined by the CNL Animal Care Committee.

### Statistical analysis

The statistical analysis was performed using One-way ANOVA with Dunnett’s multiple comparisons test using Prism Version 8 (GraphPad Software).

## Results

### LDR affects peripheral blood composition in a mouse model of spontaneous mammary gland tumorigenesis under LDR exposure

The goal of this study was to investigate the impact of chronic low-dose gamma radiation exposure on the immune status of mice with respect to the development and progression of mammary cancer after whole body radiation. To achieve this, 1.5 month-old MMTV-Neu mice that spontaneously develop mammary adenocarcinomas were exposed to continuous whole-body ^60^Co gamma-ray radiation over a period of 56 days. As a result, the mice received a total absorbed dose of 10, 100, or 2,000 mGy. Mice were sacrificed at three time points for analysis: 3.5 months of age (24 hours post-irradiation), 6 months of age (2.5 months post-irradiation), and 8 months of age (4.5 months post-irradiation) (**Figure 1**).

**Figure 2:**
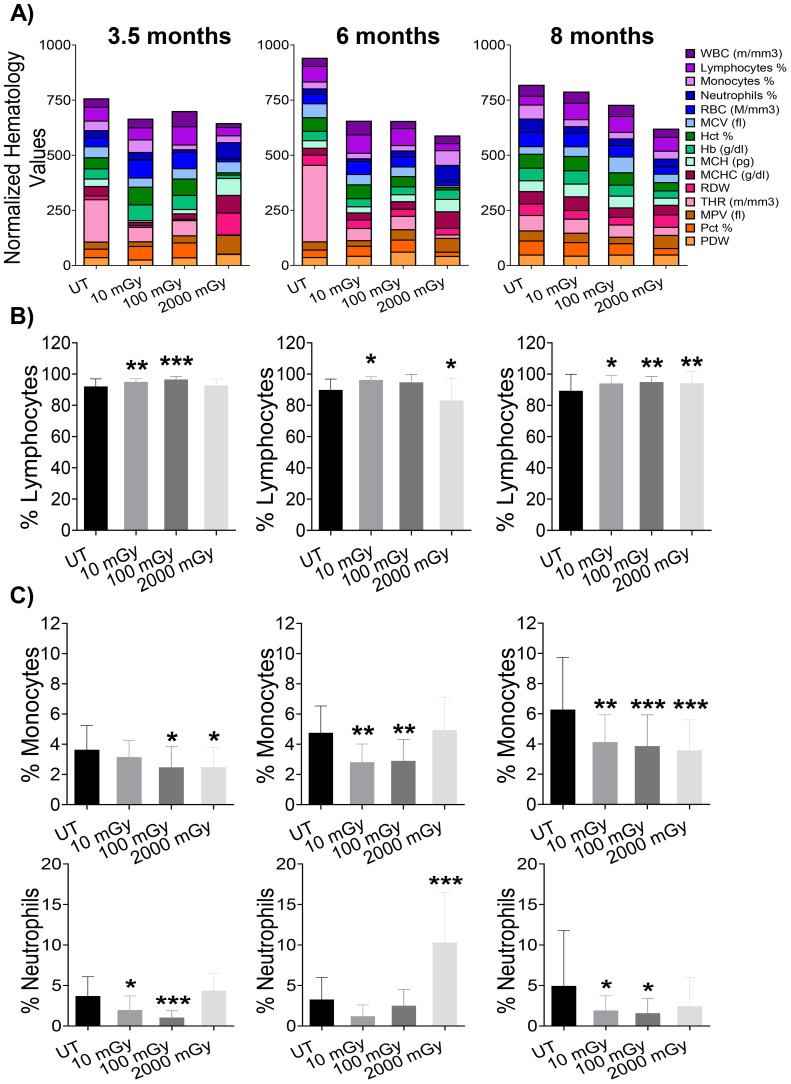
Haematology results show significant changes in blood measurements. **(A)** Bars represent normalized values for each parameter, where values are displayed as percentages (highest value = 100%; lowest value = 0%). At each designated time-points, 3.5 month (far left panel), 6 month (middle panel), and 8 month (far right panel), blood from euthanized mice was analyzed for key cell populations and protein markers. **(B, C)** Bar graphs of individual cell populations. (n 15-25); statistics were performed using One-way ANOVA with Dunnett’s multiple comparisons test; *P < 0.05; ** P < 0.01; *** P < 0.001.

Peripheral blood comprises erythrocytes and mature leukocytes that are relatively constant in mice from a particular genotype under physiological conditions, indicating a precise regulation of hematopoietic lineage differentiation and commitment (47). To assess the effects of LDR exposure on the blood components, we performed a complete peripheral blood analysis. We observed noteworthy differences in hematology metrics of blood from mice exposed to both low and high doses of radiation (**Figure 2A**), indicating that radiation exposure affected blood composition in these mice. LDR significantly, albeit minorly, increased the proportion of lymphocytes at all time points except at 6-month time-point where 100 mGy did not reach statistical significance (**Figure 2B**). On the other hand, HDR exposure showed no effect on lymphocytes at the 3.5 month time point, followed by a slight decrease in proportion at the 6 month time point (**Figure 2B**). Later, at 8 months of age, mice exposed to HDR had slightly increased lymphocyte proportions (**Figure 2B**). Generally, we observed decreased frequencies of both monocytes and neutrophils in the blood of mice following LDR exposure (**Figure 2C**). Mice exposed to HDR displayed lower monocyte frequencies at 3.5 and 8 month time points and a higher proportion of neutrophils at the 6-month time-point (**Figure 2C**). Taken together, these data suggest that radiation induces a significant impact on peripheral blood composition in a mouse model of spontaneous mammary gland tumorigenesis.

### Chronic LDR exerts a limited impact on lymphocyte proportions

**Figure 3:**
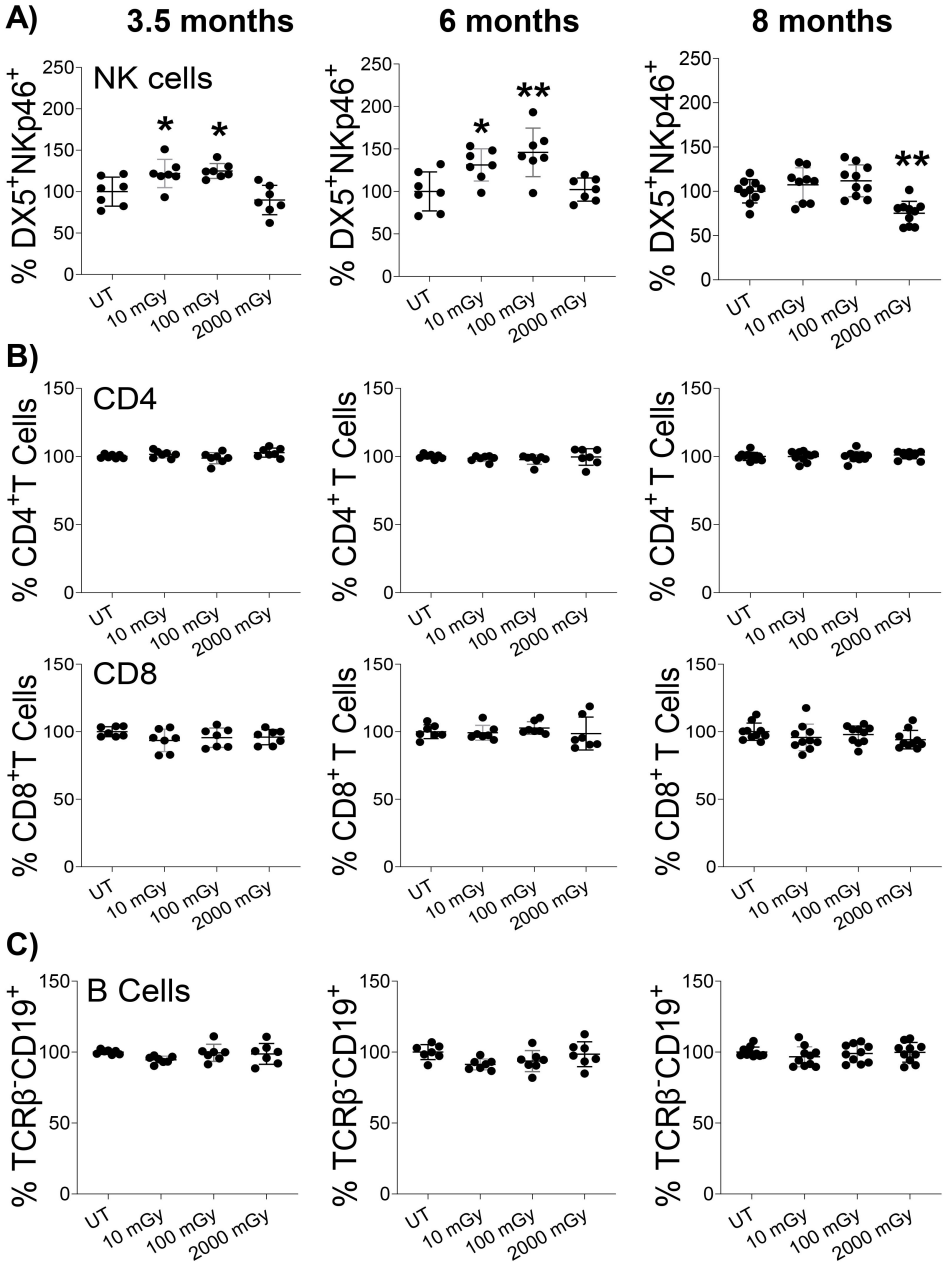
LDR increases NK cell proportion. Single-cell suspensions were generated from the spleens of mice sacrificed at the indicated time points after radiation exposure. Cells were stained with surface markers, and the relative proportion of **(A)** NK, **(B)** CD4 & CD8, and **(C)** B cells were assessed in untreated control and radiation exposed mice by flow cytometry. The y-axis represents the relative percentage of cells in the total population of mononuclear cells. The percentage of cells in untreated mice was set to 100%. (n 7~10); statistics were performed using One-way ANOVA with Dunnett’s multiple comparisons test; *P < 0.05; ** P < 0.01.

Next, we used flow cytometry to compare the proportions of different immune cells isolated from various tissues across radiation conditions as outlined in the gating strategy (**Supplementary Figure 1**). We observed an increased frequency of NK cells (TCRβ^-^ DX5^+^NKp46^+^) isolated from the spleens of mice that received LDR compared to untreated controls (**Figure 3A**). Specifically, both 10 mGy and 100 mGy radiation exposures induced elevated proportions of NK cells at the 3.5 and 6 month time points. At the 8-month time point, the NK cell proportions returned to baseline for mice exposed to LDR (**Figure 3A**). In contrast, HDR exposure (2,000 mGy) did not significantly affect the frequency of NK cells in the spleen at the early time points, however, a slight but significant reduction was measured at the 8-month time point (**Figure 3A**).

Following NK cells, we evaluated the frequencies of splenic T-cells (TCRβ^+^ NKp46^-^/DX5^-^) with respect to CD4 (TCRβ^+^ CD4^+^) and CD8 (TCRβ^+^ CD8^+^) cell subtypes. Radiation exposure did not induce any difference in the proportions of CD4^+^ T-cells and CD8^+^ T-cells at any dose or time point (**Figure 3B**). Similarly, both low and high doses of radiation did not affect the proportion of B-cells (TCRβ^-^ CD19^+^) in the spleens (**Figure 3C**). Therefore, we measured a unique but subtle modulation of NK cells in response to chronic radiation exposure. Conversely, mice exposed to HDR experienced a late reduction in splenic NK cell proportion, with NK cells maintaining baseline levels following exposure until 4.5 months post-exposure.

Furthermore, we analyzed the proportions of these immune populations in the mammary glands and lungs of irradiated mice. At 3.5 months, we observed a slight but statistically significant increase in the proportion of NK cells in the mammary glands of mice exposed to 100 mGy LDR (**Supplementary Figure 2A**). Otherwise, we measured no statistically significant impact of LDR on NK cell proportions in the mammary gland or lungs (**Supplementary Figure 2A, B**). The impact of HDR was solely significant at 8 months, when we measured a reduction in the proportion of mammary gland NK cells (**Supplementary Figure 2A**). We observed no effect of HDR on NK cell proportions in both the mammary gland and lungs (**Supplementary Figures 2A, B**). Consistent with the spleen findings, radiation did not impact the proportion of T and B cells in other examined organs (data not shown). These results highlight a limited impact of chronic LDR exposure on the frequency of NK cell populations in different analyzed organs. Importantly, our data suggests that even the lowest dose of 10 mGy could induce immunomodulation by increasing NK cell proportion.

### Chronic LDR triggers increased CD25 and NKG2D expression on NK cells

**Figure 4:**
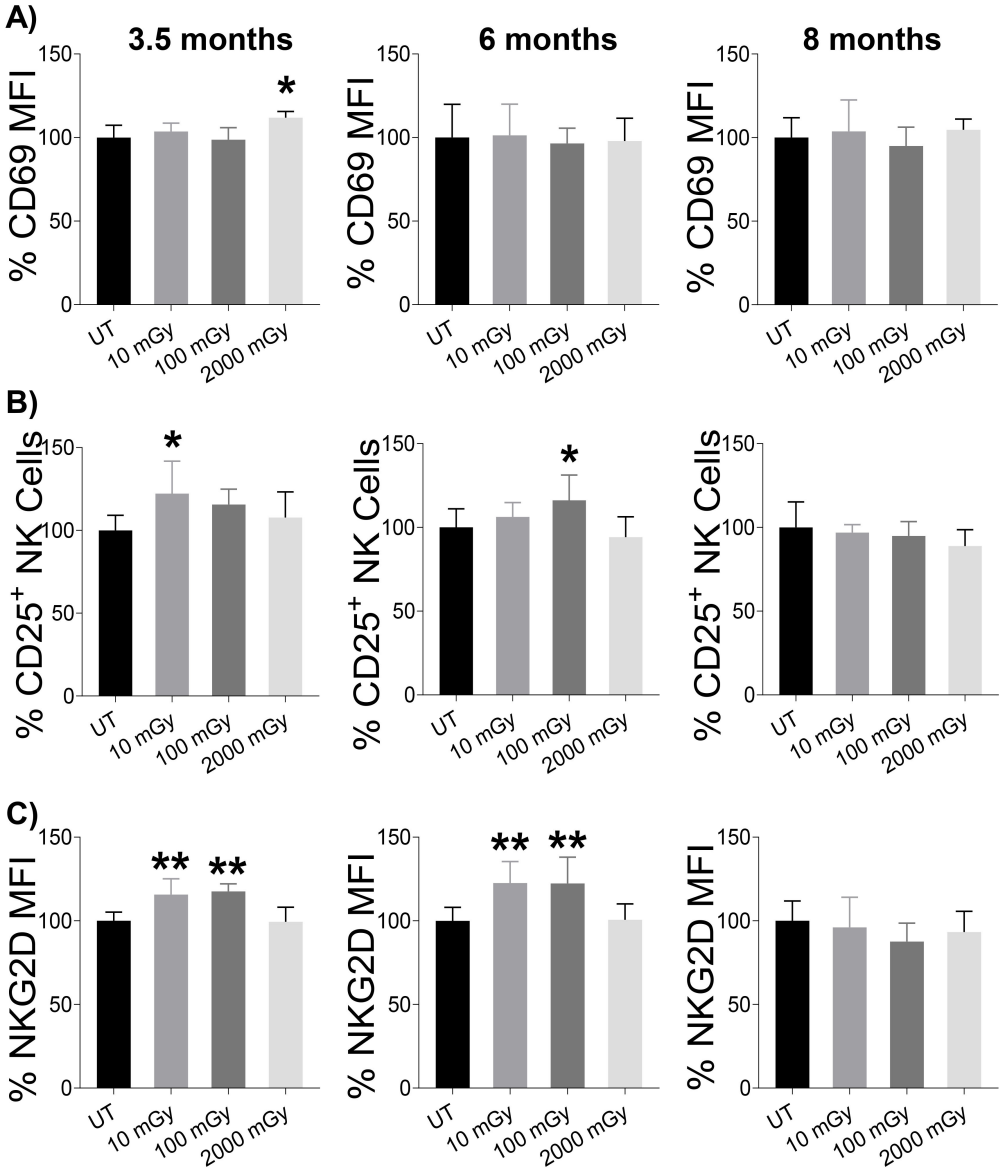
LDR-induced NK cell activation. Splenic lymphocytes from mice sacrificed at the indicated points after radiation exposure were used to measure the expression of activation markers on NK cells. **(A)** Expression of CD69 (early activation marker), **(B)** proportion of CD25^+^ cells among total population of NK cells, and **(C)** expression of NKG2D on NK cells were measured by flow cytometry after surface staining. The y-axis in (A & C) represents the relative MFI and **(B)** the relative percent of CD25^+^ cells among total NK cells. MFI and percentage cells in untreated mice were set to 100%. (n 7~10); statistics were performed using One-way ANOVA with Dunnett’s multiple comparisons test; *P < 0.05; ** P < 0.01.

With higher proportions of NK cells measured following LDR exposure, we decided to investigate whether these cells exhibited changes in activation status. Using flow cytometry, we evaluated the expression of activation-associated surface markers. First, we checked the expression of CD69, an early marker of lymphocyte activation (48). LDR did not induce the expression of CD69 on NK cells at any time point compared to untreated controls; however, increased expression was noted following HDR exposure at the 3.5-month time-point (**Figure 4A**). Consistently, the expression of CD43, a transmembrane glycoprotein expressed on activated NK cells (49), was unchanged across all groups during all three time points (**Supplementary Figure 3**). Next, we examined the expression of IL-2α receptor (CD25) that can demarcate activated NK cells (50, 51). We observed a positive trend of LDR-induced expression of CD25 on NK cells at 3.5 and 6-month time points, with 10 mGy and 100 mGy reaching statistical significance at 3.5 and 6-month time points, respectively (**Figure 4B**). This effect was not seen in mice exposed to HDR at any time point (**Figure 4B**). We also measured the expression of NKG2D, one of the activation receptors that plays a critical role in NK cell-mediated immune response to transformed cells (11, 52–55). Similarly, we found an increased expression of NKG2D with LDR at 3.5 and 6-month time points, while HDR-induced expression was not observed at any time point (**Figure 4C**). Taken together, these results indicated that LDR exposure can lead to the modulation of important NK cell activation receptors, but these effects were transient as they were not observed at 8-month time point.

### Impact of LDR on the function of immune cells

The release of cytokines and cytotoxic granules is central to immune responses. To evaluate IFNγ production, a primary cytokine produced by NK cells, splenic lymphocytes were isolated from mice in each group and were stimulated *ex vivo* with either anti-NKp46 or IL-2/IL-12 for 4 hours. We also assessed the degranulation activity by measuring lysosomal-associated membrane protein 1(LAMP-1). In order to measure the functional activity of T cells, splenic lymphocytes were stimulated with anti-CD3/28 for 16 hours, followed by intracellular staining to determine IFNγ and LAMP-1 production.

**Figure 5:**
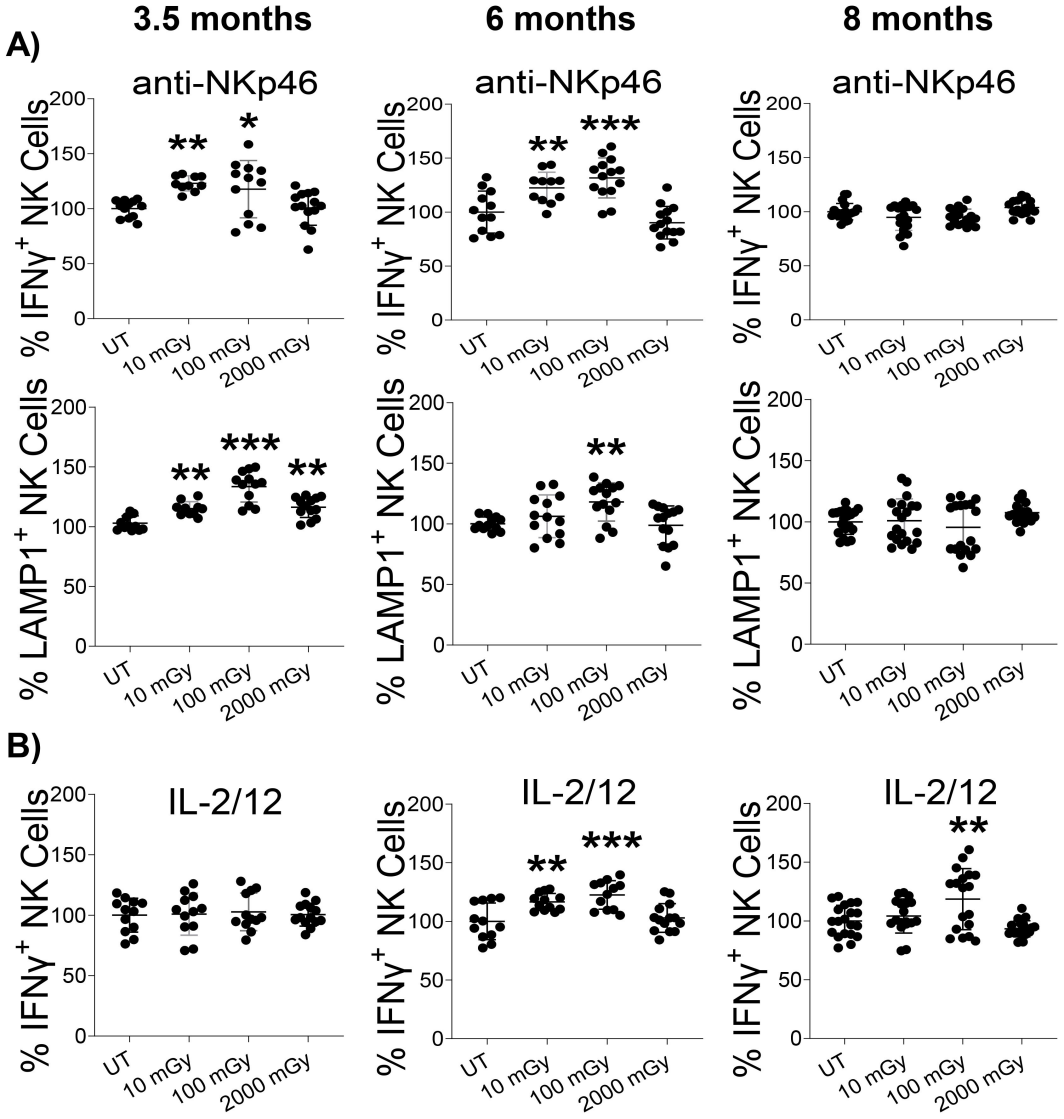
LDR augments NK cell function. Splenic lymphocytes isolated from mice sacrificed at indicated time points after radiation exposure were stimulated with either **(A)** anti-NKp46 or **(B)** IL-2 and IL-12 for 4 hours, followed by intracellular staining and flow cytometry to measure IFN-γ and CD107a (LAMP-1) positive cells among the NK cell population. The experiment was performed in duplicate. The y-axis represents the relative percentage of IFN-γ/CD107a positive NK cells. Percent cells in untreated mice were set to 100%. (n 7~10 and the experiment was conducted in duplicate); statistics were performed using One-way ANOVA with Dunnett’s multiple comparisons test; *P < 0.05; ** P < 0.01; *** P < 0.001.

We found that NK cells isolated from LDR-exposed mice at both 3.5 and 6-month time points that were stimulated with anti-NKp46 had increased proportions of NK cells producing IFNγ, compared to untreated controls (**Figure 5A**). This response was strictly observed in LDR-exposed groups, as HDR did not modulate IFNγ release. NK cells from mice exposed to any radiation condition exhibited slightly increased proportions of LAMP1-positive cells at the 3.5-month time-point. At the 6-month time point, only NK cells isolated from mice exposed to 100 mGy demonstrated increased proportions of LAMP1-expressing cells. At the 8-month time point, no effect of radiation was observed on IFNγ release or LAMP1 expression following anti-NKp46 stimulation (**Figure 5A**).

Cytokine stimulation (IL-2 and IL-12) slightly increased IFNγ production at the 6 and 8-month time points following LDR exposure, compared to untreated controls; the 10 mGy was not statistically significant at the 8-month time point (**Figure 5B**). T cells from all conditions responded similarly to anti-CD3/28 treatment, with no differences measured in the proportion of IFNγ^+^ or LAMP1^+^ T-cells (**Supplementary Figure 4**). Collectively, these results indicate that exposure to LDR can impact NK cell function by increasing IFNγ production and degranulation in response to stimulus. Notably, HDR largely did not induce any functional activity regardless of the mode of NK cell stimulation.

### Effects of LDR on the inflammatory responses

**Figure 6:**
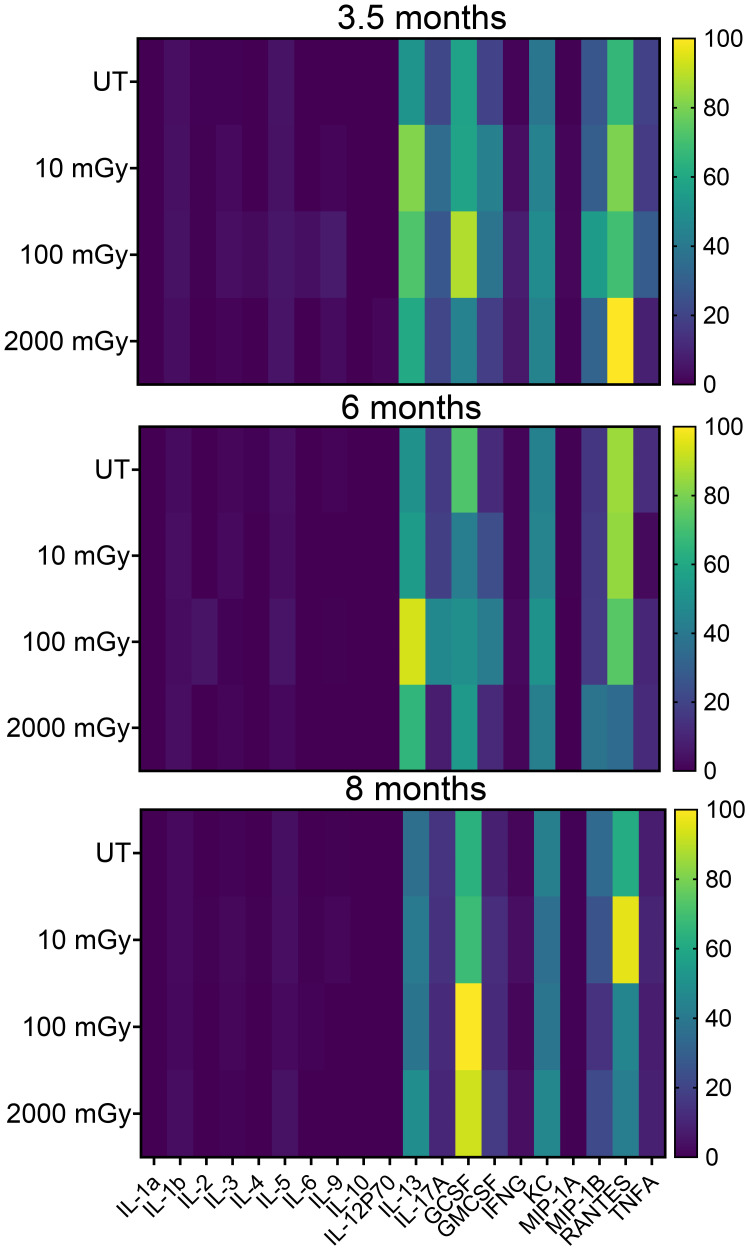
Low dose radiation promotes inflammatory conditions *in vivo*. Cytokine analysis in peripheral blood plasma with samples derived from treated and untreated MMTV-Neu mice. Concentrations were determined using the Bio-Plex HTS instrument. Heatmaps show changes in the global expression of 20 selected cytokines. (n 20~30).

Cytokines and chemokines are generally recognized as important mediators of the inflammatory response. To better understand the functional alterations of the immune system, we next analyzed the plasma levels of different cytokines in radiation-exposed and control mice. We measured a wide range of cytokines involved in anti and pro-inflammatory responses. We did not observe any noticeable difference in the plasma levels for most of the common cytokines (**Figure 6**); however, among all assessed, plasma levels of IL-13, GCSF, KC, MIP-1B RANTES were found altered in different mice groups in all time-points (**Figure 6**).

**Figure 7:**
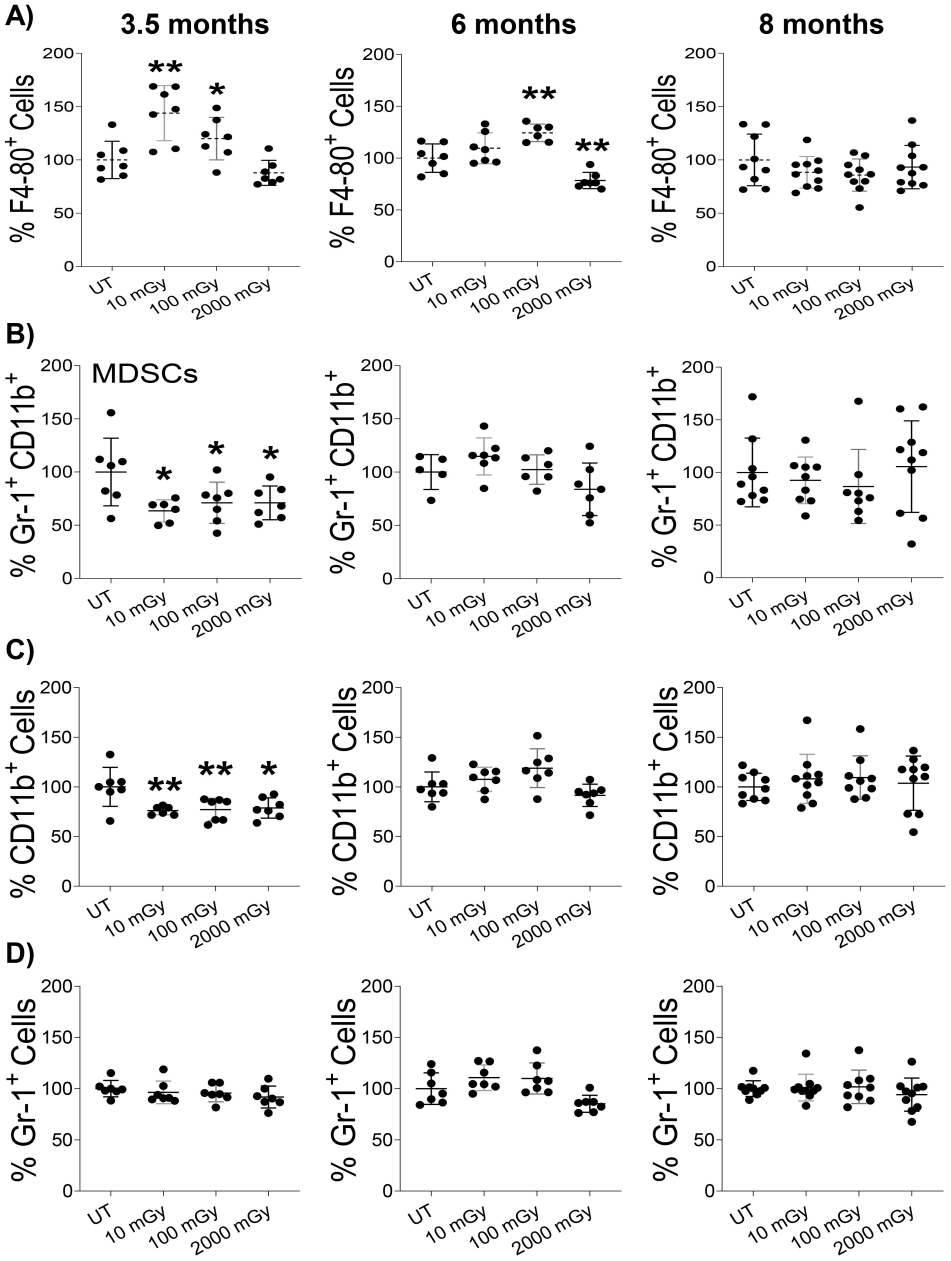
Low dose radiation promotes inflammatory conditions *in vivo* **(A–D)**. Single-cell suspension of splenic lymphocytes was stained with various surface markers to identify the proportion of inflammatory cell populations using flow cytometry in mice sacrificed at the indicated time points. The y-axis represents the relative percentage of cells in the total population of mononuclear cells. Percent cells in untreated mice were set to 100%. (n 7~10); statistics were performed using One-way ANOVA with Dunnett’s multiple comparisons test; *P < 0.05; ** P < 0.01.

To probe further, we analyzed the proportion of various sub-populations of immune cells involved in inflammation via flow cytometry by staining splenic cells for surface markers that are known to be expressed specifically on the inflammatory compartment of immune cells (56). We noticed an increased proportion of macrophages (CD45^+^F4/80^+^ cells) in the spleens of mice exposed to LDR and not HDR at the 3 and 6-month time-point, compared to untreated controls, although 10 mGy did not reach statistical significance at the 6-month time-point (**Figure 7A**). In contrast, HDR decreased the proportion of macrophages at the 6-month time-point (**Figure 7A**). Both low as well as high-dose radiation reduced the frequency of myeloid-derived suppressor cells (Gr‐1^+^CD11b^+^) at 3.5-month time-point and no differences were observed between any groups at later time points (**Figure 7B**). A similar pattern was observed in the proportion of CD11b^+^ cells, where radiation impact was only observed at 3.5 moth time point (**Figure 7C**), while radiation did not induce any differences in the proportion of Gr-1^+^ cells among all tested groups (**Figure 7D**). Notably, at the 8-month time point, we observed no effect of any radiation condition on these populations. Taken together, these results suggest differential and time-dependent effects of radiation on cytokines and immune populations involved in inflammation.

### LDR is dispensable on tumorigenesis at the organismal level

**Figure 8:**
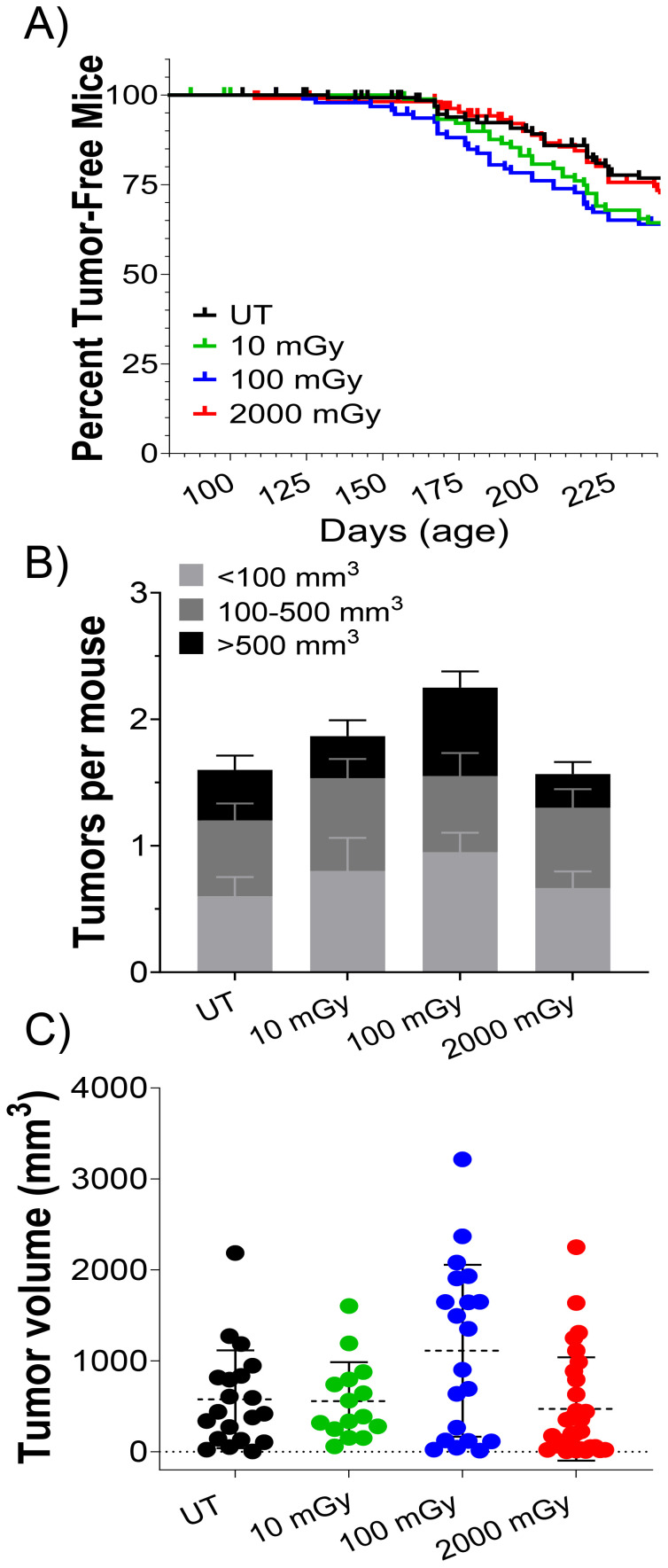
LDR affects tumor latency but not overall tumor volume and number Tumor latency in MMTV-Neu control and irradiated mice. **(A)** Kaplan Meier curve of percent of tumor-free mice plotted against days of age, censored cohorts represented by tick marks (i.e. euthanized mice; n ≈ 100 per group); Wilcoxon pairwise test showed a significant difference between UT and 100 mGy cohort in tumor latency in the Kaplan Meier curve (**A**; *P ≤ 0.05). **(B)** The number of tumors per mouse, and **(C)** tumor volume was measured at the 8-month sacrifice time point in tumor-bearing mice.

Finally, we examined the effects of LDR on the process of tumorigenesis. In MMTV-Neu mice, tumors can be easily discerned through the skin, allowing us to estimate tumor incidence and latency in all mice throughout the study (n ≈ 100 per treatment group). **Figure 8A** shows tumor latency (age at first tumor development) as percent tumor-free mice vs. time. Pairwise statistical analysis of tumor latency curves using the Log-rank Mantel-Cox test (p<0.05) showed no significant differences between any of the treatment groups. However, pairwise analysis using the Wilcoxon test, which puts greater weight on earlier time points, demonstrated a significant decrease in tumor latency in the UT vs. 100 mGy groups (**Figure 8A**). Although statistically insignificant, there was a trend of increased tumor number and volume in the low-dose cohorts (10 and 100 mGy) versus the control mice (**Figures 8B, C**). Surprisingly, mice exposed to HDR behaved close to the control group in terms of tumor burden (**Figures 8B, C**). In conclusion, our findings suggest that chronic low-dose gamma radiation may influence early tumor development, as evidenced by the significant decrease in tumor latency in the 100 mGy group, along with the observed trend towards increased tumor number and volume in low-dose cohorts. However, these effects were not consistently strong or significantly robust across all statistical analyses, indicating that the impact of low-dose radiation on tumor progression is subtle.

## Discussion

The effects of low-dose radiation on biological systems, particularly regarding cancer risk and immune modulation remain contentious. This study aimed to address this gap by investigating the impact of chronic low-dose gamma radiation on mammary tumorigenesis and immune responses in a transgenic mouse model (FVB/N-Tg(MMTVneu)202Mul/J). Notably, the overexpression of the *neu* gene in this model is specific to mammary tissue and does not affect any other physiological systems, including the immune system. By exposing these mice to continuous whole-body ^60^Co gamma-ray radiation over 56 days, we assessed the immunological and tumorigenic outcomes at various cumulative absorbed doses and follow-up points. Our findings offer valuable insights into the nuanced effects of LDR on immune cell frequency, function, and cancer progression.

One of the key findings of this study was in NK cells, where mice exposed to LDR exhibited a transient increase in their proportion, elevated activation as indicated by CD25 and NKG2D expression, and enhanced effector function in the spleen compared to controls. This increase was mainly specific to the spleen, as the mammary glands and lungs did not mirror this phenomenon. Furthermore, these effects were absent in HDR-exposed mice, underscoring the distinct immune modulation pathways activated by LDR versus HDR. This could be the reason that LDR extended the cellular longevity of NK cells through invoking DNA damage repair resulting in delayed cell death (23, 57, 58). Importantly, LDR-exposed mice displayed decreased cellular apoptosis (7, 13, 59). Therefore, it is possible that LDR could increase the lifespan of NK cells leading to their increased frequency. Murine NK cell frequency has been previously shown to be sensitive to modulation by low-dose ionizing radiation. Lacoste-Collin et al. showed that cumulative exposure to 100 mGy over a year in a lymphoma model temporally expanded splenic NK cells with no significant effect on cancer progression (60). Bogdándi et al. showed that acute doses as low as 50 mGy contract splenic NK cells (58). On the other hand, different studies have demonstrated that NK cell proportion can be radioresistant. Song et al. found that acute LDR exposure of up to 100 mGy did not modulate splenic NK cell frequency (61). Shin et al. found that low-dose chronic radiation did not alter the proportion of peripheral NK cells (62). Furthermore, liver NK cells were radioresistant to fractionated radiation at doses of 200 mGy to cumulative 800 mGy (63). In our study, we assessed the impact of LDR on NK cell proportions and activation in mice experiencing spontaneous mammary tumorigenesis. Therefore, while NK cells can be modulated in their proportion and effector function by radiation, differences in dose rate, analysis time points, mouse age, mouse strain, and mouse disease model utilized likely contribute to different outcomes across studies. Additionally, while many of the referenced studies were performed in C57BL/6 mice, our work utilized a different strain, which may itself account for some discrepancies. Factors such as the prevailing cytokine environment in the presence of tumor cells, the characteristics of the surrounding tissue microenvironment, and the tumor burden present at the time of assessment may all play important roles in shaping the observed outcomes (64).

Previous work has demonstrated that radiation can modulate the function of NK cells. Whole-body HDR has been shown to transiently increase NK cell function, however in the highest doses and later time points, HDR was associated with a functional decline (65, 66). LDR has been shown to enhance NK cell functionality *in vitro*, potentially in a P38-MAPK dependent mechanism (35, 36). Studies have shown that NK cells isolated from mice exposed to whole-body fractionated radiation have increased cytotoxicity (63, 67). Cheda et al. demonstrated that LDR could reduce sarcoma tumor engraftment in an NK cell-dependent mechanism (31). Hayase et al. observed increased cytotoxic activities of NK cells following repeated 0.5 Gy γ-irradiation (39). Same dose (0.5 Gy) exposure in mice also improved NK activity and antibody-dependent cellular cytotoxicity, resulting in delayed tumor growth (68). Combined 0.75 Gy and IL-2 treatment decreased B16F10 melanoma burden and promoted increased NK cell tumor infiltration in a mouse model (30, 69). A recent review has highlighted the impact of radiation on modulating the function of NK cells (70). Here, we provide additional evidence of a stimulatory effect of LDR on NK cells, such that NK cells isolated from mice exposed to LDR had increased IFNγ production and degranulation following stimulation.

Importantly, LDR can also change the expression of receptors on NK cells and their ligands on cancer cells, thereby affecting NK cell responses. Irradiation of breast cancer cells enhances the expression of the CXCL16 ligand, which induces the migration of natural killer cells expressing the CXCR6 receptor. Radiation exposure can upregulate a variety of NKG2D ligands on the surface of stressed cells, which can engage and modulate the expression of NKG2D on NK cells (52, 71–78). NKG2D is one of the crucial activating receptors present on NK cells, in humans and mice that can recognize many diverse ligands. The ligation of NKG2D with the corresponding ligand is sufficient to activate cytolysis and cytokine production by NK cells (55, 73). Previously, we have reported internal LDR exposure given via tritiated drinking water reduced NKG2D expression on murine NK cells, accompanied by increased NKG2D-L expression (11). Results presented in this study contradict our previous findings, indicating that NKG2D-NKG2D-L interaction upon LDR exposure is differentially regulated depending on the type of LDR exposure along with exposure methods. Along with NKG2D, we also observed higher expression of CD25, which plays a critical role in NK cell responsiveness to low doses of IL-2, leading to augmented metabolic and functional activity (51, 79). Despite increases in NK cell frequency, degranulation, cytokine production, and receptor expression, the lack of change in tumor latency and burden suggests that these changes were insufficient in mediating anti-tumor responses.

Inflammation is a part of the basic immune defense mechanism in response to harmful stimuli and can contribute to tumor suppression. We observed changes in different cytokines and chemokines involved in the inflammation processes in mice that received radiation compared to their untreated counterpart. This was consistent with previous findings indicating the role of radiation in inflammation (80) and LDR-induced regulation in a variety of inflammatory processes and pathways (81, 82). Furthermore, it has been shown that tumor-associated macrophages in irradiated tissue have enhanced secretion of pro-inflammatory cytokines (83–85), while we did not evaluate tumor-associated macrophages, we measured temporarily elevated proportions of splenic macrophages in mice exposed to LDR. Furthermore, the decreased proportion of myeloid-derived suppressor cells observed in this study may indicate reduced immunosuppressive activity, strengthening the notion that LDR can enhance immune cell function.

The impact of low-dose radiation exposures on tumorigenesis remains an active area of study (86–88). Accumulating epidemiological data indicate populations occupationally exposed to LDR may face increased cancer incidence, but whether immune modulation contributes to this risk remains unclear. Across the conditions tested, encompassing both low and high dose cumulative exposures, chronic radiation exposure did not measurably influence the overall mammary tumor latency, burden and volume in MMTV-neu mice. Modest changes in immune homeostasis measured, including those to NK cell proportion and activity, were not accompanied by altered tumorigenesis, suggesting that either the magnitude or axis of immune perturbation was insufficient to modify disease in this model. In our previous study using the same mouse strain, chronic internal low-dose radiation exposure via tritiated drinking water did not affect the tumor burden (11). Taken together, these studies highlight that both external and internal chronic radiation exert limited effects on mammary tumorigenesis in this strain.

These findings should be viewed within the context of the heterogeneous literature, where radiation has been reported to show both tumor-promoting and tumor-suppressive effects, suggesting that the LNT model may not fully account for this complexity (25, 89–92). Variability across studies likely reflects differences in (i) radiation quality, quantity and dose rate, including acute versus chronic radiation; (ii) differences in animal strains and cancer models, such as spontaneous and transplant murine cancer models; (iii) timing of irradiation; (iv) age at the time of exposure; (v) field of irradiation (92–94). For example, a previous study using rats established dose-rate and age-dependent effects of whole-body LDR on mammary carcinogenesis (89). For adult rats irradiated at a high dose rate of 60 mGy/h to achieve a total dose of 4 Gy, there was a significant increase in the hazard ratio (HR) for mammary carcinoma compared to non-irradiated controls. For dose rates between 3–24 mGy/h, the HR did not significantly increase, suggesting a threshold effect below which the carcinogenesis risk is not significant. This effect was more pronounced in juvenile rats compared to adults. A dose rate-dependent effect on mammary carcinogenesis was also observed in a study exposing BALB/c to chronic LDR (90). High-dose-rate exposures of 0.35 mGy/min to cumulative doses of 0.1, 0.2 and 0.25 Gy led to a dose-dependent increase in tumor incidence. LDR exposure of 0.1 Gy/day to 0.25 Gy total dose resulted in a lower incidence compared to high-dose rate exposures. While cross-species and cross-model generalization is inherently limited, these data are compatible with threshold-like or dose-rate–sensitive behavior in mammary carcinogenesis (92, 95). In that context, the absence of an effect in our study may reflect that our highest dose rate and/or cumulative dose remained below the regime required to perturb tumor trajectories in MMTV-neu, rather than evidence that LDR is universally without consequence.

Biologic features of the MMTV-neu system also plausibly blunt any impact of radiation-induced immune modulation. Although Neu is xenogeneic in origin, lifelong expression under the MMTV promoter in FVB/N-Tg(MMTVneu)202Mul/J mice establishes central and peripheral tolerance, functionally rendering Neu “self”. Sow et al. provide compelling evidence to that effect, showing that when Neu-expressing tumors were transplanted into wild-type hosts, they were highly immunogenic, characterized by increased tumor-infiltrating lymphocytes and spontaneous regression (96). By contrast, the same tumors transplanted into Neu transgenic mice were tolerated, permitting outgrowth. Therefore, in Neu transgenic models, the tolerance of Neu antigen restricts effective immune recognition compared to wild-type mice (96). Additionally, the tumor model may not harbor a sufficient mutational burden to generate neo-antigens, further limiting opportunities for effective immune recognition (97). In such a “cold” tumor context, perturbing immune homeostasis and or inducing radiation-induced cell death and antigen release may not translate into detectable changes in immune surveillance and tumorigenesis. Timing may further contribute. The median tumor latency in this strain is ~4 months, but our irradiation began at ~1.5 months. If early immunoediting and immune escape had already occurred by the time of exposure, subsequent immune perturbations would be less likely to re-establish control. Future studies will be needed to determine how radiation influences immune function across tumors of varying immunogenicity.

In a clinical context, this work has implications for many individuals, in particular women who may be exposed to risk factors associated with the development the Her2 positive cancer subtype. Routine mammograms and employment as nuclear energy workers would increase the radiation exposure and lifetime dose for these women. The typical mammogram delivers a dose of 3–5 m Gy per screening (98). Clinical studies conducted in the United States have suggested that radiation-induced cancers from digital mammograms are rare and are estimated to be 0.4–1.2 per 10,000 women screened over a lifetime (99). Epidemiological studies examining nuclear energy workers (NEWs) is far more complex, as radiation dose, dose rate, and source are important considerations. Recently published work examining NEWs from five Department of Energy (DOE) sites in the United States, Hanford site, Idaho National Laboratory, Oak Ridge National Laboratory, and Savannah River Site, examined solid cancer incidents from workers who had been employed for at least one year [84]. Exposures ranged from 0 to 1109 mSv, skewed to lower exposures as the median equivalent dose was 4 mSv. Out of the 101,363 workers examined, 19,564 (19%) were female. Of this group and restricted to employees with cumulative exposures <200 mSv, the excess relative risk of developing breast cancer was negative (-)2.39 (100). As the correlation between radiation exposure (<200 mSv) and breast cancer was negative, this finding suggests that low-dose radiation may have a protective effect. It should be noted, however, that the subtype of breast cancer was not reported in the study.

Publications that examined the correlation of low-dose radiation exposure and specific breast cancer subtypes are lacking. This is likely due to information regarding subtypes not being available or recorded for NEWs that developed breast cancer, and as women have historically been underrepresented in the nuclear field, there are likely insufficient numbers in each subtype to observe trends and conduct impactful analysis. In conclusion, this study is important in understanding the effects of gamma rays on the innate and adaptive immune response in the context of breast cancer progression, specifically in the notoriously aggressive Her2-positive tumors. This work lends to future experiments examining the potential role of low-dose radiation on different breast cancer subtypes.

In summary, we witnessed LDR-induced potent effects on the proportion, activation, and function of immune cells. Notable changes in tumor latency were observed between the 100 mGy cohort and control when weighted to earlier time-points, however, limited changes were observed at later time-points. Ultimately, our data opens exciting new avenues for future cancer therapies by taking advantage of the potential of low-dose radiation to boost systemic immunity.

## Conclusion

This study contributes to understanding the impact of chronic low-dose ionizing radiation on the immune system in the context of breast cancer progression. In summary, our results showcase the modest immunomodulatory effects of chronic low-dose gamma radiation, highlighting its distinct impact on NK cell proportion and function and its mixed effects on inflammatory cytokines and cell populations. These LDR-induced immune changes, however, had a minimal impact on mammary tumorigenesis, with only slight reductions in tumor latency and minor increases in tumor number and volume observed. Overall, the effects of low-dose radiation on tumor development are subtle, indicating a limited influence on cancer progression.

## Data Availability

The original contributions presented in the study are included in the article/**Supplementary Material**. Further inquiries can be directed to the corresponding author/s.
