# Supplementary figures and images for: The impact of low-dose gamma radiation on immune modulation in a mouse model of spontaneous mammary gland tumorigenesis
Source: Front Immunol. 2025 Nov 3;16:1635779. doi: 10.3389/fimmu.2025.1635779 (PMC12620402; doi:10.3389/fimmu.2025.1635779)

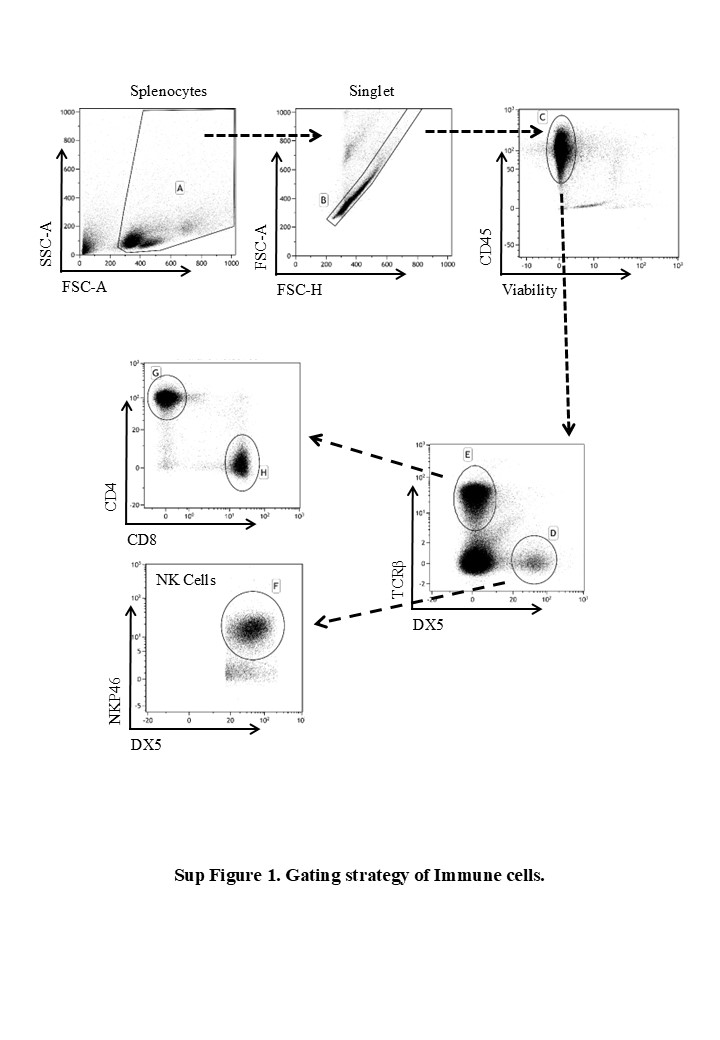

Supplement: Supplementary file 1 [file Image1.jpeg]

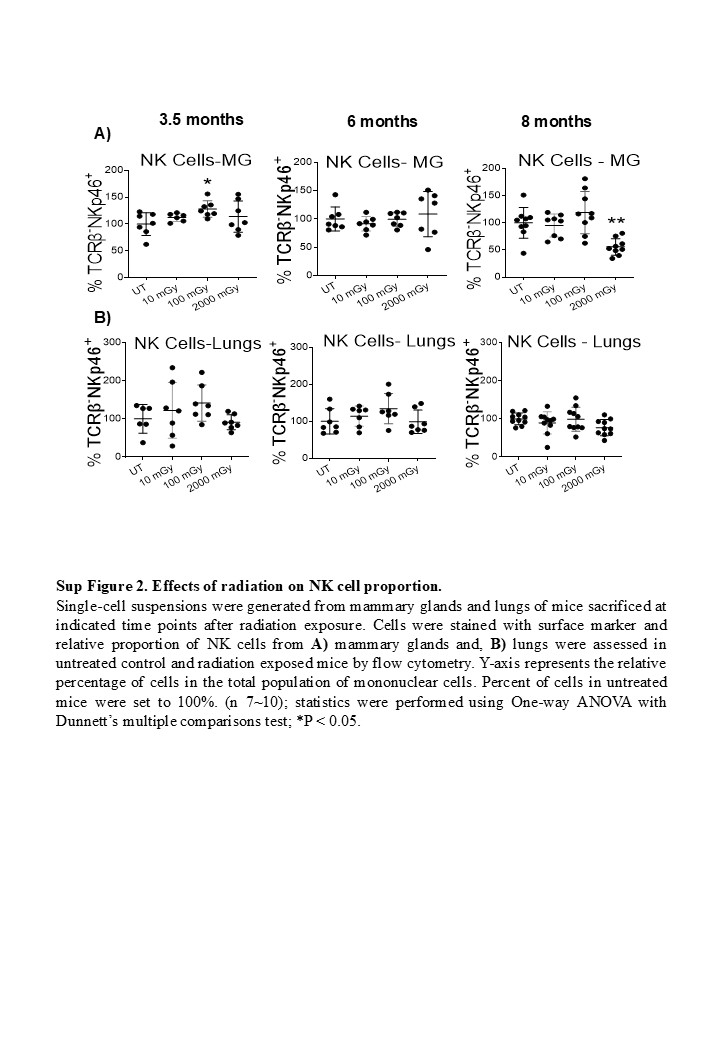

Supplement: Supplementary file 2 [file Image2.jpeg]

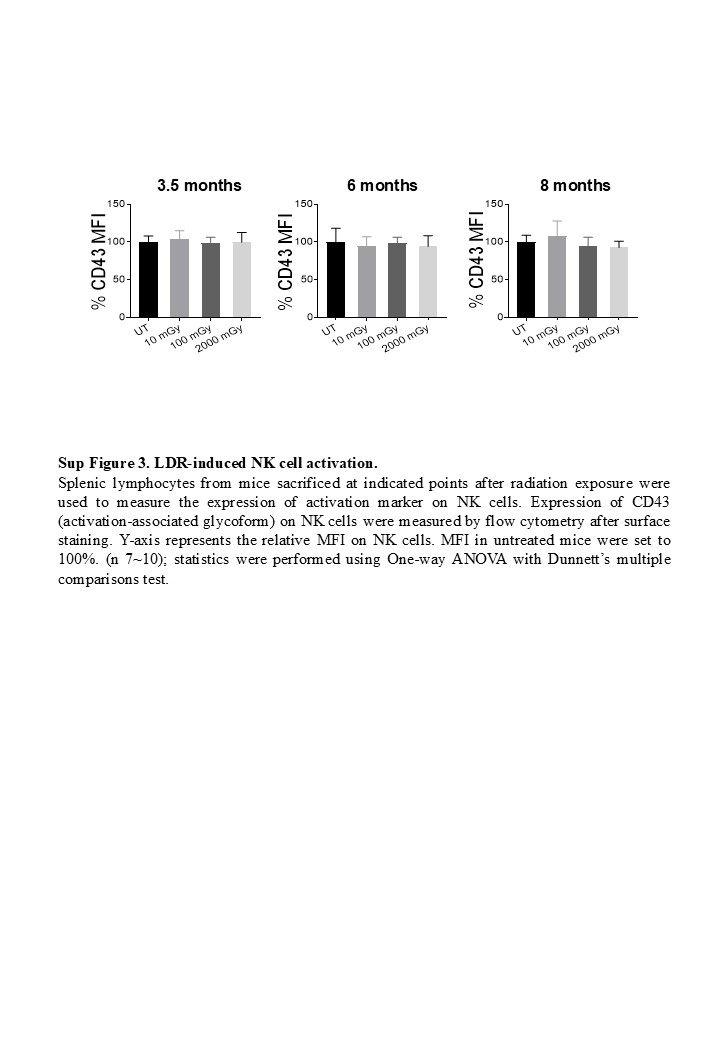

Supplement: Supplementary file 3 [file Image3.jpeg]

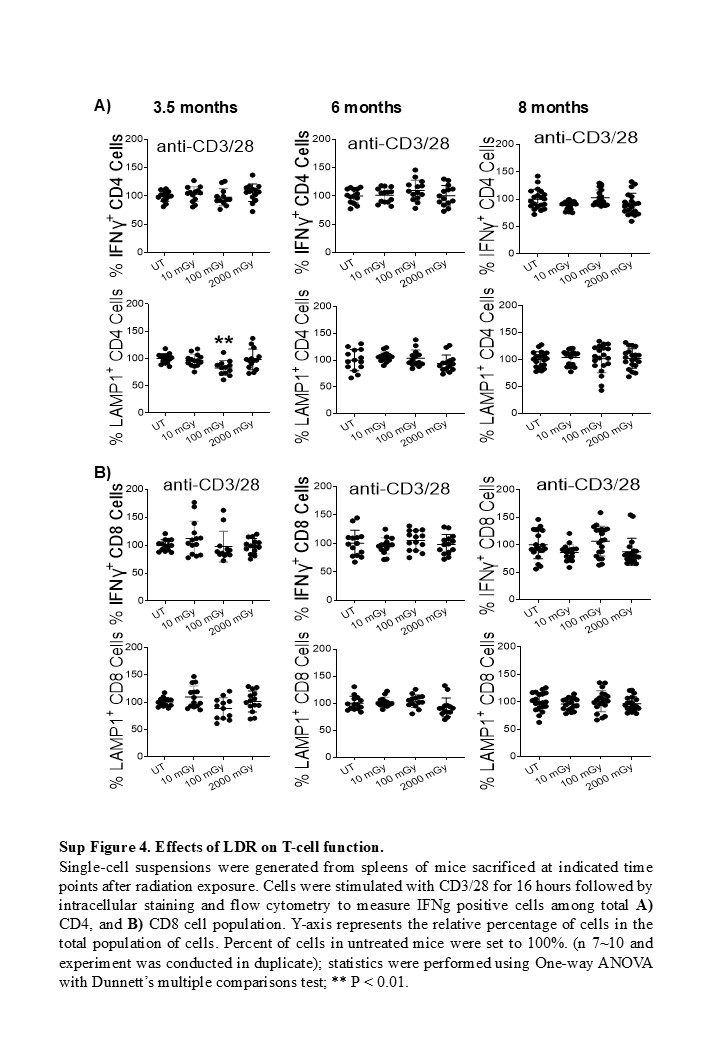

Supplement: Supplementary file 4 [file Image4.jpeg]
